# Endoparasite Infections of the European Hedgehog (*Erinaceus europaeus*) in Central Italy

**DOI:** 10.3390/ani11113171

**Published:** 2021-11-06

**Authors:** Alessia Mariacher, Andrea Santini, Irene Del Lesto, Sara Tonon, Elena Cardini, Antonino Barone, Claudia Eleni, Gianluca Fichi, Stefania Perrucci

**Affiliations:** 1 Istituto Zooprofilattico Sperimentale delle Regioni Lazio e Toscana, 58100 Grosseto, Italy; alessia.mariacher@izslt.it (A.M.); andrea.santini-esterno@izslt.it (A.S.); irene.dellesto-esterno@izslt.it (I.D.L.); elena.cardini91@gmail.com (E.C.); 2 Istituto Zooprofilattico Sperimentale delle Regioni Lazio e Toscana, 00178 Roma, Italy; sara.tonon-esterno@izslt.it (S.T.); claudia.eleni@izslt.it (C.E.); 3 Istituto Zooprofilattico Sperimentale delle Regioni Lazio e Toscana, 01100 Viterbo, Italy; antonino.barone@izslt.it; 4 Dipartimento di Scienze Veterinarie, Università di Pisa, 56124 Pisa, Italy

**Keywords:** *Brachylaemus erinacei*, *Capillaria erinacei*, *Crenosoma striatum*, *Cystoisospora rastegaievae*, *Physaloptera clausa*, hedgehog, wildlife

## Abstract

**Simple Summary:**

The European hedgehog is a very common animal in Europe. This animal usually lives in woods but often it is possible to find it in gardens and parks in urban areas. Few studies describe parasites infecting the European hedgehog in Italy. In the present study, endoparasite infections and associated lesions were investigated in hedgehogs from two different regions of central Italy (Latium and Tuscany), where no investigations had been performed before. Several helminth and protozoan species were identified in the intestine and respiratory tract of the animals analyzed. The presence of the respiratory worm *Crenosoma striatum* was found to be associated with bronchial lesions. This study contributed to the knowledge of parasitic infections of the European hedgehog, the most frequently hospitalized animal in wildlife rescue centers.

**Abstract:**

The European hedgehog is a synanthropic mammal, widely distributed in Europe. This species usually inhabits the edges of deciduous or mixed woods, but it is also very common in private gardens and public parks. Despite its popularity and frequency of contacts both with humans and with wild and domestic animals, few studies have examined the endoparasitic fauna of the hedgehog in Italy. In the present study, endoparasites of naturally deceased hedgehogs (*n* = 40) from central Italy (Latium and Tuscany regions) were investigated, along with concurrent gross and histopathological lesions. The most prevalent identified endoparasites were *Crenosoma striatum* (45%), *Capillaria erinacei* (42.5%) and *Brachylaemus erinacei* (22.5%), in accordance with previous reports from hedgehogs in southern Italy. In few subjects, *Physaloptera clausa*, Acanthocephalans and *Cystoisospora* *rastegaeivae* coccidia were also identified. The infection by the lungworm *C. striatum* was found to be significantly associated (*p* < 0.01) with bronchial hyperplasia and peribronchiolitis upon histopathological examination. Awareness of the most common parasitic infections in the hedgehog and of their effects on the health of these animals is extremely important, especially in wildlife rescue centers, where European hedgehog represents the most frequently hospitalized mammal species.

## 1. Introduction

The European hedgehog, *Erinaceus europaeus*, is a nocturnal synanthropic mammal, widely distributed in Europe [1,2]. In Italy, the European hedgehog is a protected wildlife species under the Bern convention (included in Appendix III of Bern convention) and national law 157/1992. The hedgehog population is abundant and there is no evidence of massive decline, and therefore it is assessed as a Least Concern (LC) species by the European Mammal Assessment (IUCN 2008).

The natural habitat of this species is represented by the edges of deciduous or mixed woods, but it is also very common in suburban and rural areas, mainly in gardens and public parks [3]. For this reason, it is a very popular animal, which encounters a positive attitude on the part of humans [1].

The foraging area of the European hedgehog usually extends over a radius of 200–300 m around the nest, but it can also cover distances of several kilometers, with a considerable risk of being killed by vehicles or dying of unnatural causes [3,4,5,6,7]. As further proof of the close contact between hedgehogs and humans, this species is one of the most hospitalized mammal species in wildlife rescue centers in Italy [8] and other European countries as well [9,10,11,12,13,14,15].

Hedgehogs are omnivorous in diet, and they mainly feed on invertebrates, such as slugs, earthworms, beetles, caterpillars and other insects. These invertebrates can act as intermediate or paratenic hosts for several parasites of the hedgehog, which can cause morbidity or mortality in this species, for instance *Physaloptera clausa*, *Crenosoma striatum*, *Brachylaemus erinacei* or *Hymenolepis erinacei* [16,17]. Additionally, some parasite species recorded in the hedgehog may show a zoonotic potential, such as *Cryptosporidium* spp. [2,18], while other parasites, such as *Physaloptera clausa*, may act as vectors for human pathogens such as *Leptospira* spp. [19].

The ecological and feeding habits of the European hedgehogs, along with their high population densities, synanthropic attitudes and common contacts with both wild and domestic animals, implicate the potential involvement of this species in the maintenance and spread of parasites with zoonotic potential or also infecting other wild and domestic animal species, such as *Eucoleus aerophilus* [2,20,21,22]. Despite this and the popularity of the hedgehog, few studies have explored the endoparasite fauna of the hedgehog in Italy [1,23,24,25], compared to other European countries. The aim of this study was to investigate the endoparasite fauna of hedgehogs from two different regions of central Italy (Latium and Tuscany), where no investigations had been reported before.

## 2. Materials and Methods

### 2.1. Animals

Between January and August 2021, 40 hedgehogs (*Erinaceus europaeus*), found dead in Central Italy, were necropsied. The hedgehogs in this study were either provided by wildlife rescue centers or were collected as victims of predation (usually by domestic dogs) or road traffic accidents. All specimens were in good conservation conditions. After the collection of fecal samples, the carcasses were frozen at −20 °C, and later thawed overnight at room temperature before the analyses were carried out. At necropsy, subjects were weighed, and the sex and age class were determined. Animals were classified as juveniles or adults based on physical development, bodyweight and season of finding.

### 2.2. Parasitological Examination

Selected organs (lungs with bronchi and trachea, stomach and intestine) were cut open and examined by a dissecting microscope for the presence of adult parasites. Recovered adult helminths were examined soon after collection or preserved in 70% ethanol until identified at the species level based on their morphologic and metric features [16,22,26,27].

Individual fecal samples were qualitatively examined. Approximately two grams of feces were homogenized in 10 mL of water and filtered through gauze. The obtained fecal homogenate was centrifuged in a 15 mL tube for 5 min at 1600× *g*, and the sediment was examined by the flotation test by using a zinc sulphate (ZnS) solution with a specific gravity of 1.350. Fecal samples positive for coccidia were dissolved in 2.5% K_2_Cr_2_O_7_ solution at 22 ± 1 °C and checked daily until sporulation of oocysts, that were examined for evaluating their morphometrical features. Fecal smears from the intestinal content were also performed and stained by Kinyoun’s acid-fast method for *Cryptosporidium* spp. detection [28].

The identification of the parasitic stages found at parasitological examination of fecal samples (eggs, larvae and oocysts) was based on morphological and metrical features [27,29,30].

### 2.3. Gross and Histopathological Analysis

Internal organs were examined for the presence of gross lesions. Regardless of the presence of infection and/or macroscopically evident lesions, tissue samples from the lung, stomach and intestine were fixed in 10% neutral buffered formalin, embedded in paraffin wax, sectioned at 4 μm, stained with Hematoxylin and Eosin (HE) and examined for histopathological lesions.

### 2.4. Data Analyses

All statistical analyses were conducted in R Language [31]. Fisher’s exact test was used for testing the association between parasitic infection and histopathological lesions. Differences were considered statistically significant when the *p*-value was less than 0.05. Prevalence and the 95% confidence interval (95% CI) were also calculated.

## 3. Results

### 3.1. Animals

The 40 deceased hedgehogs examined in this study were identified as 22 males and 18 females, and 15 juveniles and 25 adults. Twenty-one animals were collected after natural death from Centro Recupero Fauna Selvatica Lipu, Rome, Latium (41°54′ 56″ N, 12°29′00″ E), eight from Parco Regionale Riviera di Ulisse, Latina, Latium (41°15′22″ N, 13°42′18″ E), four from Giardino Faunistico Pian dell’Abatino, Rieti, Latium (42°14′16″ N, 12°50′10″ E), three from Riserva Naturale “Lago di Vico”, Viterbo, Latium (42°19′05″ N, 12°10′09″ E), and four from Grosseto, Tuscany (42°48′59″ N, 11°08′07″ E).

### 3.2. Parasitological Analysis

From the parasitological examination of the respiratory and digestive tract and fecal samples, 22 hedgehogs out of 40 resulted positive for almost one parasite, with an overall infection prevalence of 55%.

**Figure 1:**
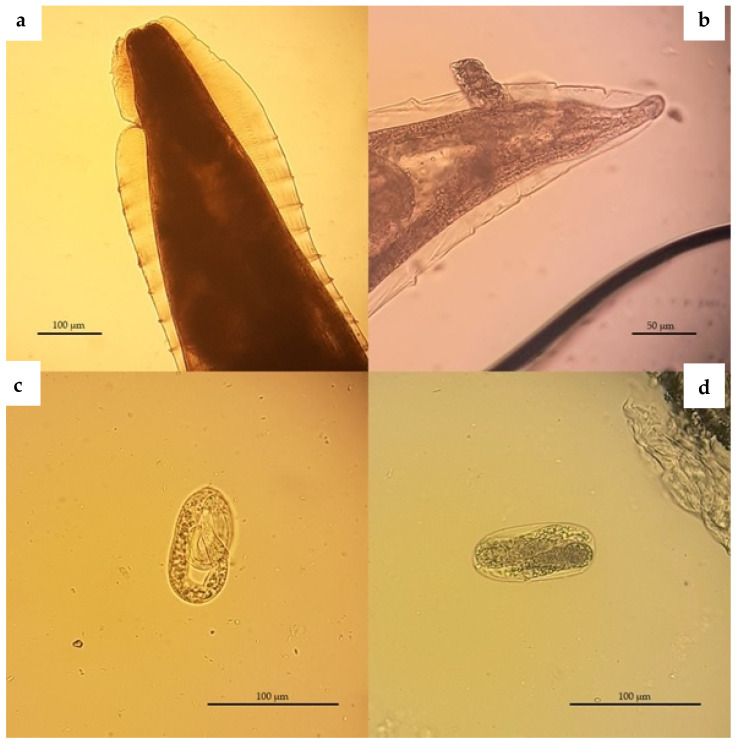
European hedgehog. *Crenosoma striatum*, adults and larvated eggs isolated from lung tissue. (**a**) Anterior end, body cross-striations of the cuticle, (**b**) posterior extremity of the female and (**c**,**d**) eggs containing first-stage larvae.

**Figure 2:**
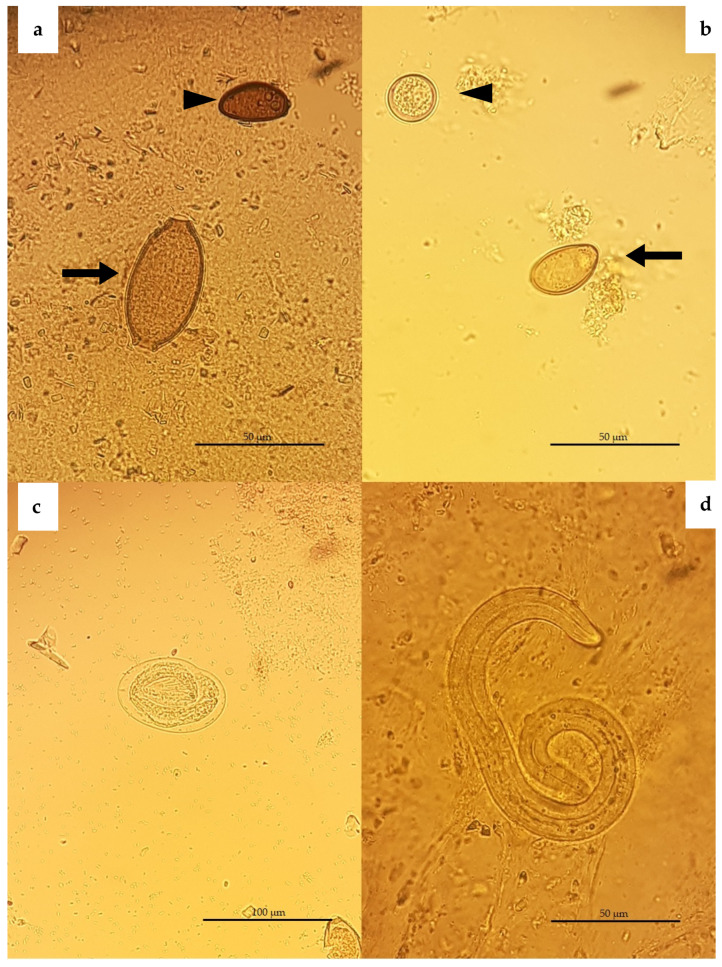
European hedgehog, flotation test with zinc sulphate solution (specific gravity of 1.350). (**a**) Arrow: *Capillaria erinacei* egg with bipolar plugs, measuring 55.37 µm *×* 27.29 µm on average; Arrowhead: *Brachylaemus erinacei* egg, measuring 32.18 µm × 22.91 µm. (**b**) Arrowhead: *Cystoisospora rastegaievae* oocyst, mean size 19.12 µm × 18.23 µm; Arrow: *Brachylaemus erinacei* egg. (**c**) *Physaloptera clausa* egg, mean 42.92 µm × 28.78 µm. (**d**) *Crenosoma striatum*, first-stage larva of 285 µm × 16 µm in size.

**Figure 3:**
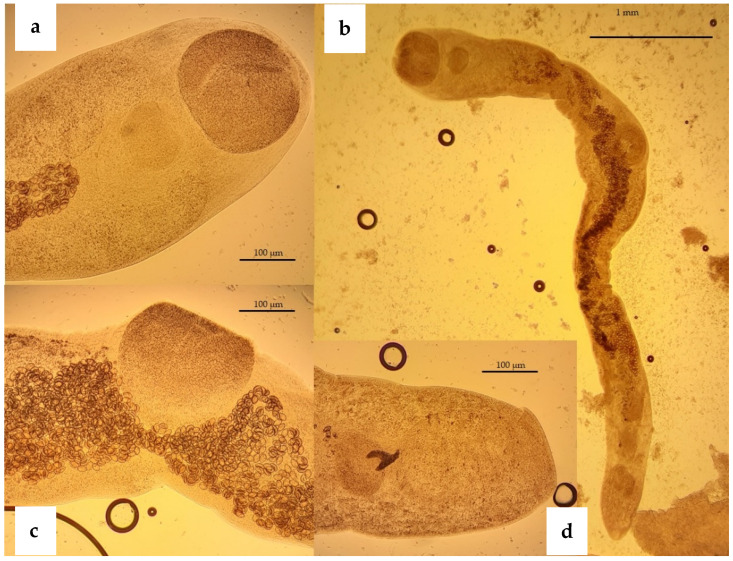
European hedgehog. *Brachylaemus erinacei*, adults isolated from the small intestine. (**a**) Oral sucker of 229.7 µm in diameter, (**b**) adult specimen measuring 2.85 mm in length, (**c**) ventral sucker of 185.4 µm in diameter and eggs measuring 32.18 µm × 22.91 µm and (**d**) caudal end with male and female gonads.

In total, one protozoan and five helminth species, including three nematodes, one trematode and one Acanthocephalan, were found. The most prevalent species was *Crenosoma striatum* (45%; 95% CI 29.58–60.42) (Figure 1 and Figure 2b), followed by *Capillaria erinacei* (42.5%; 95% CI 27.2–57.8) (Figure 2a) and *Brachylaemus erinacei* (22.5%; 95% CI 9.6–35.4) (Figure 2a,b and Figure 3), while a lower prevalence was observed for *Cystoisospora rastegaivae* (5%) (Figure 2b), *Physaloptera clausa* (2.5%) (Figure 2c) and Acanthocephala (2.5%).

Among positive hedgehogs, only 18.2% (4/22) of animals were found infected by a single parasite species, while multiple parasite species were identified in most infected hedgehogs (18/22, 81.8%). Specifically, two different parasites were observed in thirteen animals (59.1%), three in four animals (18.2%) and four in one animal (4.5%). The highest prevalence was observed in male (59.1%) and adult (80%) hedgehogs, while juveniles showed a very low prevalence (13.3%), with only two hedgehogs being positive for *B. erinacei* and *C. striatum,* respectively. No statistical difference was observed between male and female prevalence, while the prevalence in adults was statistically higher (*p* < 0.01) than in juveniles, especially for *C. striatum* and *C. erinacei*. All examined fecal smears were negative for the presence of *Cryptosporidium* spp. oocysts.

The prevalence and 95% confidence interval for each parasite found in the different sex and age groups are reported in Table 1. Characteristic features of identified eggs or adult parasites are shown above in Figure 1, Figure 2 and Figure 3.

### 3.3. Gross and Histopathological Analysis

**Figure 4:**
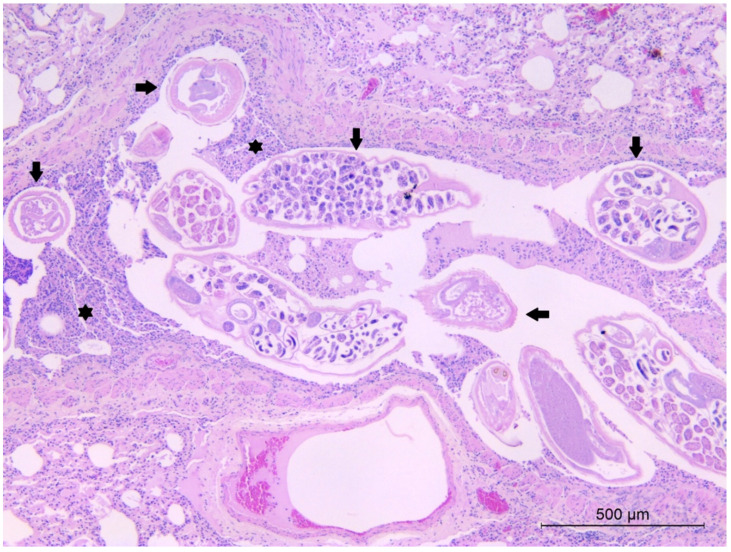
European hedgehog, lung. Sections of adult *C. striatum* nematodes in the lumen of a bronchus (arrows), associated with hyperplasia (asterisks) of the bronchial epithelium. Hematoxylin and Eosin (HE).

At necropsy, gross pulmonary lesions were rarely observed in both lungworm-infected and not infected hedgehogs. The majority of lesions were consistent with road traffic accidents (hemorrhages and lacerations). In three subjects found heavily infected by *C. striatum*, bundles of adult nematodes protruded from the cut surface of lungs at necropsy. Histological examination of lungs in *C. striatum*-infected animals showed the presence of worms in the lumen of the bronchi and bronchioles in 13 out of 18 animals (72.2%), along with hyperplasia of the bronchial epithelium (Figure 4) and mild to moderate inflammation. Inflammatory infiltrate consisted of lymphocytes, plasma cells and few eosinophils and neutrophils. Mild chronic peribronchitis and peribronchiolitis were also frequently observed, while granulomatous foci, with macrophages and multinucleated cells, were only occasionally detected in the lung parenchyma. Overall presence of lung lesions at histological examination was higher in *C. striatum*-infected animals compared to uninfected subjects (*p* < 0.05), with a higher significance for bronchial hyperplasia and peribronchiolitis (*p* < 0.01) (Table 2).

**Figure 5:**
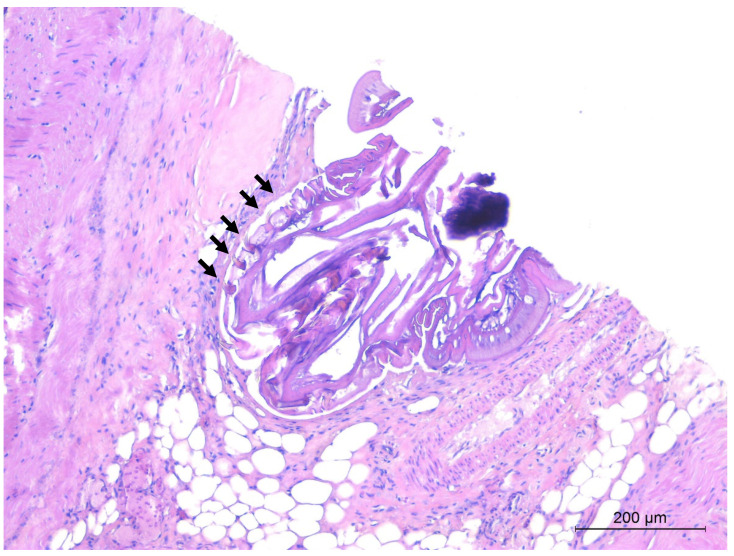
European hedgehog, intestine. Section of proboscis with spines (arrows) of an adult Acanthocephalan parasite, embedded in the intestinal mucosa. Hematoxylin and Eosin (HE).

Sections of worms and nematode eggs were frequently observed microscopically in the lumen of the intestine, usually not associated with significant gross or histopathological lesions in the intestine wall. In some cases, a mild inflammatory infiltrate, consisting of lymphocytes, plasma cells and rare eosinophils, was detected in the lamina propria of the intestinal mucosa. In a single sample, a section of an adult Acanthocephalan parasite was evidenced (Figure 5), while the respective coprological sample was negative for the presence of Acanthocephalan eggs.

## 4. Discussion

The prevalence of infected animals in the present study (55%) is lower than in studies conducted both in Italy (100%) [1,24] and in other countries worldwide, such as Iran (95%) [17], Ireland (91%) [32], the United Kingdom (91%, 90%) [22,33], Spain (90.4%) [34] and Greece (79%) [35]. However, in several studies, only adult animals were examined [1,24], while in the present study, juveniles were also included. The adult age class was confirmed here to be the one with the highest prevalence of parasite infections (80.0%), compared to younger animals (*p* < 0.01). No hoglets (very young hedgehogs under 100 g in body weight) were examined in the present study, but in the available literature, there is agreement upon this age class being generally not frequently positive for parasites [17,32]. Gaglio and colleagues [22] suggested that, since parasite accumulation is age-dependent, this could provide a plausible explanation for older individuals being generally more heavily infected with parasites. However, no significant variation between the age and parasitic load of hedgehogs was observed in other studies [31,36].

The most prevalent species in this study was *C. striatum*, which is a known cause of verminous pneumonia in hedgehogs. The observed prevalence in this study (45%) is consistent with data from Portugal [16] and Greece [30], but lower compared to previous Italian studies [1,24], which reported prevalence rates of over 60% (Table 3). *C. striatum* is a meta-strongyloid nematode characterized by an indirect lifecycle, with the hedgehog as the final host. Animals acquire infection by ingestion of third-stage larvae contained in intermediate hosts such as snails and slugs [22,25,37,38]. Infected hedgehogs may present with a range of clinical signs such as weight loss, nasal discharge, dyspnea, wheezing, cough and exercise intolerance [16,22,37,38,39,40]. Verminous pneumonia may frequently result in a fatal disease for hedgehogs, also due to secondary bacterial infections with *Bordetella bronchiseptica* or *Pasteurella multocida*, lung abscessation and pleuropneumonia [38]. After treatment, irreversible lung damage may persist, with pulmonary consolidation [38]. Lung lesions of crenosomosis in the hedgehog were previously described by Hoseini and colleagues [41], and more recently by Barradas and colleagues [16]. In the present study, we investigated the statistical association between *C. striatum* infection and the presence of lung lesions upon histopathological examination, which was highly significant for bronchial hyperplasia and peribronchiolitis (*p* < 0.01). However, more animals should be analyzed to draw definitive conclusions on *C. striatum* pathogenicity.

In heavily infected individuals, bundles of whitish nematodes protruded from the cut surface of the lower airways at necropsy. Only *C. striatum* adults were identified among respiratory nematodes, while no *Eucoleus aerophilus* was identified [38,40,42]. In Europe, *E. aerophilus* infection seems to occur less frequently than *C. striatum* infection, with reported prevalence rates of under 40% in various countries (Table 3). In Italy, *E. aerophilus* was reported in the hedgehog only by Manzocchi and colleagues [25], while it was not identified in all other previous parasitological reports [1,24].

**Table 3:** Presence and prevalence data from the literature on parasites of the European hedgehog (*Erinaceus europaeus*) in different European countries. Number of positive animals or prevalence were calculated if not directly reported in the original studies. Reports describing *Capillaria* sp. eggs in feces, with no distinction made between *Eucoleus aerophilus* or gastrointestinal capillariid eggs, were excluded.

	Parasite Species	Site of Infection	Country	Number of Positive/Number of Examined (Prevalence %) [Reference]
NEMATODA	*Crenosoma striatum*	Trachea, bronchi and alveolar ducts	Czech Republic	19/71 (26.4) [43]
			Germany	88/133 (66.4) [44] 55/205 (26.8) [45]
			Great Britain	17/39 (43.6) [36] 30/42 (71.4) [40] 10/74 (13.5) [46] 52/74 (71.0) [22] 37/47 (79.0) [47]
			Greece	9/19 (47.4) [35]
			Ireland	7/7 (100.0) [32]
			Italy	30/39 (76.9) [24] 54/87 (62.1) ^a^ [1] 1/1 [25]
			Portugal	5/11 (45.5) [16]
			Spain	73/117 (62.4) [34]
			United Kingdom	21/30 (70.0) [44]
	*Haemonchus contortus*	Stomach	Italy	1/34 (2.9) [1]
	*Eucoleus aerophilus* (syn. *Capillaria aerophila*)	Trachea, bronchi, bronchioles	Czech Republic	19/71 (26.4) [43]
			Germany	54/133 (40.9) [44]
			Great Britain	23/74 (32.0) [22]
			Italy	1/1 [25]
			Poland	1/15 (6.7) [3]
			United Kingdom	8/30 (26.7) [44]
	*Capillaria* sp. (ova and/or adults in trachea, bronchi or lungs)		Great Britain	8/8 (100.0) [40] 1/13 (8.0) [36]
	*Capillaria erinacei* (syn. *Aonchotheca erinacei*)	Stomach and small intestine	Great Britain	30/39 (79.0) [36]
			Ireland	19/22 (86.0) [32]
			Italy	12/39 (30.7) [24] 49/87 (56.3) [1]
			Poland	9/15 (60.0) [3]
			Spain	52/125 (41.6) [34]
	*Capillaria* sp. (adults in small intestine)	Small intestine	Czech Republic	54/72 (75.0) [43]
			Germany	109/133 (81.8) [44]
			Great Britain	13/74 (17.6) [46] 45/74 (61.0) [22]
			United Kingdom	27/30 (90.0) [44]
	*Capillaria* sp. (adults in stomach)	Stomach	Great Britain	48/74 (66.0) [22]
	*Eucoleus tenuis*		Spain	2/117 (1.7) [34]
	*Gongylonema* sp.	Esophagus	Italy	17/39 (43.5) [24]
	*Spirura rytipleurites*	Stomach	Italy	12/39 (30.7) [24] 10/34 (29.4) [1]
			Spain	27/125 (21.6) [34]
	*Physaloptera clausa*	Stomach	Czech Republic	2/72 (2.8) [43]
			Greece	6/19 (31.6) [35]
			Italy	1/34 (2.9) [1]
			Spain	8/125 (6.4) [34]
	*Pterygodermatites plagiostoma* (syn. *Rictularia plagiostoma*)		Spain	1/125 (0.8) [34]
TREMATODA	*Trematoda* (eggs in feces)		Greece	2/19 (10.5) [35]
	*Brachylaemus erinacei* (syn. *Brachylaima erinacei*)	Small intestine	Czech Republic	42/72 (58.3) [43]
			Germany	8/168 (4.8) [18] 46/133 (34.4) [44]
			Great Britain	2/74 (2.7) [46] 40/74 (55.0) [22] 1/47 (2.1) [47]
			Italy	16/39 (41.0) [24] 24/87 (27.6) [1]
			Poland	5/15 (33.3) [3]
			Spain	48/125 (38.4) [34]
			United Kingdom	16/30 (53.3) [44]
	*Brachylecitum aetechini*	Liver	Italy	1/39 (2.5) [24]
	*Brachylecithum mackoi*	Liver	Italy	1/1 [21]
	*Dicrocelium dendriticum*	Liver	Italy	1/39 (2.5) [24]
CESTODA	*Hymenolepis erinacei* (syn. *Vampirolepis erinacei*, *Rodentolepis erinacei*)	Small intestine	Czech Republic	1/72 (1.4) [42]
			Greece	1/19 (5.3) [35]
			Great Britain	1/74 (1.4) [46] 1/13 (8.0) [36]
	*Mesocestoides* (larvae)		Italy	3/39 (7.6) [24] 1/34 (2.9) [1]
ACANTHOCEPHALA	*Acanthocephala* (eggs in feces)		Greece	3/19 (15.8) [35]
	*Nephridiorhyncus major* (syn. *Nephridiacanthus major*)	Intestine and gut serosa	Czech Republic	3/71 (4.2) [43]
			Italy	27/39 (69.2) [24] 8/34 (23.5) [1]
			Spain	1/125 (0.8) [34]
	*Oliganthorhynchus erinacei* (syn. *Echinorhynchus erinacei*)	Intestine and gut serosa	Great Britain	13/74 (18.0) [22]
	*Plagiorhyncus cylindraceus*	Intestine and gut serosa	Germany	21/133 (16.1) [44]
			Czech Republic	4/72 (5.6) [43]
			United Kingdom	8/30 (26.7) [44]
	*Prostorhyncus* sp.	Omentum	Great Britain	2/74 (2.7) [46]
			Spain	5/125 (4.0) [34]
PROTOZOA				
	*Isospora rastegaivae*	Intestine	Italy	1/39 (2.5) [24]
	Coccidian	Intestine	Greece	3/19 (15.8) [35]
	*Ooocysts* (not differentiating between *Isospora* or *Eimeria*)	Intestine	Germany	35/168 (20.8) [18]
			Great Britain	2/47 (4.3) [47]
	*Giardia* sp.	Intestine	The Netherlands	10/90 (11.0) [48]
	*Cryptosporidium* sp.	Intestine	Germany	56/188 (29.8) [18]
			Great Britain	9/111 (8.0) [49]
			The Netherlands	8/90 (9.0) ^b^ [48]
	*Cystoisospora* spp.	Intestine	Germany	27/205 (14.1) [45]

^a^ Data from Giannetto et al. (1993) were included in the study by Poglayen et al. (2003) [1], but extracted for the purposes of this table. ^b^ Two genospecies, *C. parvum* (subtype: IIaA17G1R1 and IIcA5G3) and *C. hominis* (subtype: IbA10G2), were observed.

Other capillariid nematodes have been more frequently reported in hedgehogs, such as *C. erinacei* from the stomach and small intestine. In the present study, *C. erinacei* infection was only observed in adult animals, with a prevalence of 68.0%, which is higher than in previous Italian reports but in line with that found in other European countries (Table 3). *C. erinacei* infection was not associated with significant lesions of the intestinal wall in this study, and only in a few cases was a mild mixed inflammatory infiltrate detected. It is possible that only in case of heavy parasitic load may gastroenteric lesions become significant, as reported by Sainsbury and colleagues [50]. Severe infections can also cause clinical signs such as lethargy, weight loss and diarrhea [51].

Differently from *C. erinacei*, which seems to be rarely pathogenic, acute eosinophilic gastritis or chronic gastritis with atrophy of glands and fibrosis have been described in association with *Physaloptera clausa* infection [26,39,52,53]. Prevalence of this parasite in our study was low (2.5%), similarly to some previous Italian reports [1]. Higher prevalence rates for *P. clausa* have been reported in Greece (Table 3), and in other countries and in *Erinaceus concolor*, the southern white-breasted hedgehog [34,39,52,54].

*B. erinacei* is an intestinal fluke frequently reported in the hedgehog and considered responsible for severe lesions and clinical signs in this animal [55]. The infection is acquired by the ingestion of snail intermediate hosts [43]. Symptoms caused by *B. erinacei* include excessive weight loss, restlessness and diarrhea, often containing blood [56]. The infection can prove lethal, especially in young hedgehogs. However, death can also be observed in adult animals in high-load infections, which can lead to hemorrhagic enteritis, anemia and secondary bacterial infections [57].

Data obtained in this study confirm the low prevalence of Acanthocephalan infection in the hedgehog reported by previous studies [34,43,46]. Indeed, Acanthocephalans in hedgehogs are seldom reported and infections may be under-recorded, despite the fact that some Acanthocephalan species may cause major damage to the host intestinal lining, via the armed proboscis invading the intestinal wall [58,59]. The Acanthocephalan species infecting the single positive hedgehog in this study was not identified, as it was found only at histopathological examination. However, *Nephridiacanthus major* (syn. *Nephridiorhyncus major*) is a species frequently reported for infecting the hedgehog worldwide, including in Italy [1,24,60]

*C. rastegaievae* is considered a common protozoan species infecting hedgehogs, and it may contribute to the development of clinical signs such as dark-green droppings and hemorrhagic enteritis in hedgehogs of all ages [56,61].

## 5. Conclusions

In conclusion, data from this study evidenced a high prevalence of multiple endoparasite infections in *E. europaeus* from central Italy. Included among prevalent parasites were species considered highly pathogenic for the hedgehog, such as *C. striatum*. Lung lesions associated with this parasite were also highlighted in this study based on pathological findings. Awareness of parasitic infections in hedgehogs is particularly important in wildlife rescue centers [49], both to thoroughly assess the health status of rehabilitating animals and to prevent the spread of pathogens to other wild or domestic animal species in the area of release.

## Figures and Tables

**Table 1:** Identified parasites in European hedgehogs from central Italy (*n* = 40), with respective prevalence (*p*) and 95% confidence interval (CI). ^A,B^: *p* < 0.01.

	Males (*n* = 22)			Females (*n* = 18)			Adults (*n* = 25)			Juveniles (*n* = 15)			Total (*n* = 40)		
	*n*	*p* %	95% CI	*n*	*p* %	95% CI	*n*	*p* %	95% CI	*n*	*p* %	95% CI	*n*	*p* %	95% CI
Total infected hedgehogs	13	59.1	38.55–79.64	9	50.0	26.90–73.10	20	80.0 ^B^	64.32–95.68	2	13.3 ^A^	0.0–30.5	22	55.0	39.58–70.42
*Crenosoma striatum*	9	40.9	20.36–61.45	9	50.0	26.90–73.10	17	68.0 ^B^	49.71–79.20	1	6.7 ^A^	0.0–19.3	18	45.0	29.58–60.42
*Capillaria erinacei*	11	50.0	29.11–70.89	6	33.3	11.56–55.11	17	68.0 ^B^	49.71–86.29	0	0.0 ^A^	-	17	42.5	27.2–57.8
*Brachylaemus erinacei*	7	31.8	12.35–51.28	2	11.1	0.00–25.63	8	32.0	13.71–50.29	1	6.7	0.0–19.3	9	22.5	9.6–35.4
*Physaloptera clausa*	1	4.5	0.00–13.25	0	0.0	-	1	4.0	0.00–11.68	0	0.0	-	1	2.5	0.0–7.34
Acanthocephalan	0	0.0	-	1	5.6	0.00–16.14	1	4.0	0.00–11.68	0	0.0	-	1	2.5	0.0–7.34
*Cystoisospora rastegaievae*	2	9.1	0.00–21.10	0	0.0	-	2	8.0	0.00–18.36	0	0.0	-	2	5.0	0.0–11.7

**Table 2:** Comparison between presence of pulmonary and bronchial histological lesions in *C. striatum*-positive and -negative hedgehogs. ^A,B^: *p* < 0.01; ^a,b^: *p* < 0.05.

	*C. Striatum*-Positive (*n* = 18)	*C. Striatum*-Negative (*n* = 22)
Overall presence of lung lesions	15 ^a^	10 ^b^
Granulomatous lesions	1	0
Fibrinous pneumonia	4	1
Interstitial pneumonia	3	3
Pulmonary oedema	1	6
Bronchial epithelium hyperplasia	6 ^A^	0 ^B^
Peribronchiolitis with lymphocytes and plasma cells	6 ^A^	0 ^B^
